# Carnosic Acid (CA) Induces a Brown Fat-like Phenotype, Increases Mitochondrial Biogenesis, and Activates AMPK in 3T3-L1 Adipocytes

**DOI:** 10.3390/biomedicines12071569

**Published:** 2024-07-15

**Authors:** Filip Vlavcheski, Rebecca E. K. MacPherson, Val Fajardo, Newman Sze, Evangelia Tsiani

**Affiliations:** 1 Department of Health Sciences, Brock University, St. Catharines, ON L2S 3A1, Canada; 2 Centre for Bone and Muscle Health, Brock University, St. Catharines, ON L2S 3A1, Canada

**Keywords:** obesity, metabolic syndrome, adipocytes, browning, AMPK, carnosic acid

## Abstract

Adipose tissue plays a crucial role in regulating metabolic homeostasis, and its dysfunction in obesity leads to insulin resistance and type 2 diabetes (T2D). White adipose tissue (WAT) primarily stores energy as lipids, while brown adipose tissue (BAT) regulates thermogenesis by dissipating energy as heat. The process of browning involves the transdifferentiation of WAT into brown-like or beige adipocytes, which exhibit a similar phenotype as BAT. The browning of WAT is an attractive approach against obesity and T2D, and the activation of the energy sensor AMP-activated protein kinase (AMPK) has been shown to play a role in browning. Carnosic acid (CA), a polyphenolic diterpene, found in many plants including rosemary, is reported to possess potent antioxidant, anti-inflammatory, and anti-hyperglycemic properties. The limited evidence available indicates that CA activates AMPK and may have anti-obesity and antidiabetic potential; however, the effects in adipocyte browning remain largely unexplored. This study aimed to examine the effects of CA on the markers of adipocyte browning. The treatment of 3T3L1 adipocytes with CA activated AMPK, reduced lipid accumulation, and increased the expression of browning protein markers (UCP-1, PGC-1α, PRDM16, and TFAM) and mitochondrial biogenesis. The use of compound C, an AMPK inhibitor, significantly attenuated the effects of CA, indicating AMPK involvement. These studies demonstrate that CA can activate AMPK and stimulate the browning of white adipocytes. Future animal and human studies are required to examine the effects of CA in vivo.

## 1. Introduction

Obesity is the accumulation of excess body fat mostly due to an imbalance between energy intake and expenditure [1,2,3]. According to a World Health Organization (WHO) report, there are currently 1.25 billion people classified as overweight, with a body mass index (BMI) between 25.0 to 29.9 kg/m^2^, and 650 million as obese (BMI > 30 kg/m^2^) [4]. Obesity significantly increases the incidence of comorbidities, including cardiovascular and metabolic diseases, insulin resistance, and type 2 diabetes mellitus (T2DM) [5]. Hence, it is imperative that researchers find ways to reduce obesity and combat the associated excess in adiposity.

According to their distinct origin, tissue distributions, and metabolic functions, adipose tissue depots can be grouped into three types: white, brown, and beige [6]. White adipose tissue (WAT) predominantly stores energy in the form of lipids, while brown adipose tissue (BAT) consumes energy. One mechanism in which brown adipocytes dissipate the stored energy/lipids is through the activation of uncoupling protein-1 (UCP-1), which uncouples the production of adenosine triphosphate (ATP) from mitochondrial respiration, inherently processing/combusting high levels of lipids through β-oxidation [7,8]. Beige or brite adipocytes are an inducible form of adipocytes found within WAT depots that share similar cellular morphology and functions (i.e., the uncoupling of the mitochondrial proton gradient) with BAT [9]. The induction of beige adipocytes can be initiated when WAT is exposed to thermogenic stimuli such as cold, exercise, certain antidiabetic medications such as metformin, and phytochemicals in a process called “browning” [9]. The browning of white adipocytes is often correlated with a reduction in lipid accumulation, as energy substrates, i.e., lipids, are required to generate heat; thus, the browning process itself is considered an attractive strategy against obesity given the relatively low abundance of BAT in humans and considerably high amount of WAT in conditions such as obesity. Mechanistically, cold exposure, metformin, and other various compounds have been shown to induce browning via the activation of AMPK and, thus, the stimulation of signaling molecules involved in browning, including UCP-1, PRD1-BF1-RIZ1 homologous domain-containing 16 (PRDM16), peroxisome proliferator-activated receptor γ (PPARγ), and mitochondrial biogenesis such as peroxisome proliferator-activated receptor-gamma coactivator (PGC)-α and mitochondrial transcription factor A (TFAM) [9]. In addition, recently, it was demonstrated that glycogen synthase kinase 3 (GSK3) negatively regulates BAT-based thermogenesis, and the activation of AMPK, in turn, inhibits its activity [10]. Therefore, AMPK may be used as a potential target for browning and obesity.

Carnosic acid (CA) is a polyphenolic diterpene, abundantly found in rosemary extract (RE), shown to display antioxidant, anti-inflammatory, and anticancer properties [11,12]. Previous research from our lab has shown that RE [13] and its polyphenolic compounds CA [14], rosmarinic acid (RA) [15,16], and carnosol (COH) [17] can all activate AMPK, leading to enhanced glucose uptake in muscle cells. In addition, we also recently found that treatment with 10 μM CA for 24 h attenuated palmitate-induced insulin resistance in muscle and fat (3T3-L1) cells—a result accompanied by an increase in AMPK activation [18]. Corresponding well with this, there has been a recent emergence of studies indicating that CA may exhibit anti-obesity and anti-diabetic effects in animal models of obesity and insulin resistance [12,19,20,21]. However, whether this can be attributed to, at least in part, to an activation of AMPK and an induction of adipocyte browning remains unknown. In the present study, we examined the effects of CA on AMPK signaling, adipocyte browning, and mitochondrial biogenesis in 3T3-L1 cells.

## 2. Materials and Methods

Fetal bovine serum (FBS), dimethyl sulfoxide (DMSO), palmitate, bovine serum albumin (BSA), carnosic acid, metformin, cytochalasin B, glutamine, 3-Isobutyl-1-methylxanthine (IBMX), dexamethasone, rosiglitazone, CL 316 243, Oil Red O, AlexaFlour488 (CAT A-21206), PRDM16 (PA5-20872), and PGC-1α (ab3242) were purchased from Sigma Life Sciences (St. Louis, MO, USA). Materials for cell culture and trypan blue solution 0.4% were purchased from GIBCO Life Technologies (Burlington, ON, USA). Phospho- and total ACC (CAT 2661 and 3662, respectively), AMPK (CAT 2531 and 2532, respectively), PPARγ (CAT 2430S), TFAM (7495S), β-actin (4970S), and HRP-conjugated anti-rabbit antibodies (CAT 7074) were purchased from New England BioLabs (NEB) (Mississauga, ON, Canada). Anti-UCP-1 rabbit polyclonal antibodies (CAT ab10983) and compound C were purchased from Abcam (Boston, MA, USA). Insulin (Humulin R) was obtained from Eli Lilly (Indianapolis, IN, USA). MitoTracker (CAT M22426) and Hoechst (H21486) stain was purchased from Invitrogen (Waltham, MA, USA). Luminol Enhancer reagents, polyvinylidene difluoride (PVDF) membrane, reagents for electrophoresis, and Bradford protein assay reagent were purchased from BioRad (Hercules, CA, USA). [3H]-2-deoxy-D-glucose was purchased from PerkinElmer (Boston, MA, USA).

Cell Culture: Wild-type 3T3-L1 adipocytes (obtained originally from Dr A Klip’s lab and used in our lab the last 5 years) were grown in Dulbecco’s Modified Eagle Medium (DMEM) containing 10% (*v*/*v*) FBS and 2 mM glutamine (basal media). At confluence, the cells were exposed to differentiation induction medium (DMEM containing 10% FBS, 0.5 mM IBMX, 0.25 μM dexamethasone, 1 μg/mL insulin, and 2 μM rosiglitazone) for 4 days, followed by exposure to a second differentiation medium (DMEM containing 10% FBS and 1 μg/mL insulin) for an additional 3 days. A CA stock solution of 100 mM dissolved in DMSO was used to treat all cells. Prior to each experiment, a working stock of 1 mM CA was prepared in DMEM media. All treatments were performed using serum-free media. In a previous study by our group, we found a significant increase in 3T3-L1 adipocyte glucose uptake and the activation of AMPK with 10 μM CA for 24 h [18], and this concentration and duration was used in the present study. The concentrations of metformin (MET) (5 mM), used to treat the cells, were chosen based on previous studies conducted in 3T3-L1 mouse adipocytes [22,23].

Immunoblotting: After treatment, the cells were rinsed with cold PBS and lysed using cold lysis buffer containing 150 mM NaCI, 1mM sodium orthovanadate (Na_3_VO_4_), 1% Triton X-100, 20 mM Tris (PH 7.5), 1 µg/mL leupeptin, 1 mM ethylene glycol-bis β-aminoethyl ether/egtazic acid (EGTA), 1 mM ethylenediaminetetraacetic acid (EDTA 1 mM phenylmethylsulfonyl fluoride (PMSF), 2.5 mM sodium pyrophosphate, and 1 mM p-glycerolphosphate. The lysates were collected, and the protein concentration was determined using the Bradford assay. The lysates were mixed with 5% β-mercaptoethanol-containing SDS buffer and placed in boiling water for 5 min, followed by sodium dodecyl sulfate-polyacrylamide gel electrophoresis (SDS-PAGE) for protein separation and transfer to a PVDF membrane. The membranes were exposed to blocking buffer 5% (*w*/*v*) dry milk powder in Tris-buffered saline for 1 h, followed by overnight exposure, at 4 °C, to the primary antibody against the protein of interest, followed by secondary HRP-conjugated anti-rabbit antibody for 1 h and exposure to ECL substrate (ClarityMax). The Western blot bands were visualized using the ChemiDoc software version 3.0.1 (Hercules, CA, USA) and analyzed using ImageJ (imagej.nih.gov, accessed on January 2019–October 2021). The results are presented as the ratio of the density of the target phosphorylated protein to the density of the total target protein in arbitrary units, where each value is made relative to its control.

Immunocytochemistry: Fully differentiated adipocytes were fixed with 4% paraformaldehyde for 15 min, washed three times with PBS, and permeabilized and blocked with PBS containing 0.3% Triton X-100 and 3% BSA for 45 min. The cells were then incubated with anti-UCP-1 antibody (diluted 1:300) at 4 °C overnight, rinsed with PBS, and incubated with Alexa Fluor secondary antibody (1:500) for 90 min at room temperature protected from the light. Hoechst staining for the nuclei was also performed. Cell images were detected with a fluorescence microscope Cytation Gen5 imaging Microscope (BioTek, Winooski, VT, USA) (×0 objective lens) using the GFP-Green and DAPI filters to visualize the mitochondria and nuclei, respectively.

MitoTracker Staining: To explore changes in mitochondrial density, after treatment, fully differentiated adipocytes were exposed to serum-free DMEM media containing 200 nM MitoTracker Red probe and Hoechst nuclear stain (Invitrogen, USA), followed by incubation at 37 °C for 40 min. A fluorescence Cytation Gen5 Imaging Microscope (×20 objective lens) utilizing the Texas Red and Hoechst filters was used to visualize the mitochondria and nuclei, respectively. Images were quantified using ImageJ and are expressed in arbitrary units.

Oil Red O Staining: After treatment, fully differentiated 3T3-L1 adipocytes were washed three times with PBS and fixed with 4% paraformaldehyde for 15 min at room temperature. The cells were stained with a 0.5% filtered Oil Red O (ORO) solution in 60% isopropanol for 1 h at room temperature, followed by a PBS wash three times. The cells were then visualized using the Color field filter on a Cytation Gen5 imaging Microscope (×20 objective lens). The incorporated ORO dye was solubilized with 100% isopropanol and the intensity of the red was measured at 490 nm using an Cytation Gen5 ELISA plate reader. The intensity of the red is directly proportional to the amount of lipid accumulated in the cells.

Cell Viability Assay: 3T3-L1 lipoblasts or fully differentiated adipocytes were treated without (control, C) or with CA for 24 h. After treatment, the cells were incubated in 100 μL of fresh serum-free DMEM media containing 5 mg/mL MTT for 4 h. Respiring/viable cells convert MTT (3-(4,5-dimethylthiazol2-yl)-2,5-diphenyltetrazolium bromide) to a purple formazan product. The amount of formazan is proportionate to the number of viable cells. The accumulated dye was then solubilized with DMSO, the absorbance was measured at 570 nm, and the results are expressed as a percent of the untreated controls.

Statistical Analysis: Statistical analysis was performed using the GraphPad Prism software 5.3 manufactured by Graphpad Software Inc. (La Jolla, CA, USA). The data from 3–7 independent experiments were pooled and are presented as the mean ± standard error (SE). An analysis of variance (ANOVA) followed by Tukey’s post hoc test for multiple comparisons was used for statistical analysis.

## 3. Results

### 3.1. Carnosic Acid (CA) Reduces Lipid Accumulation in Mature 3T3-L1 Adipocytes

**Figure 1:**
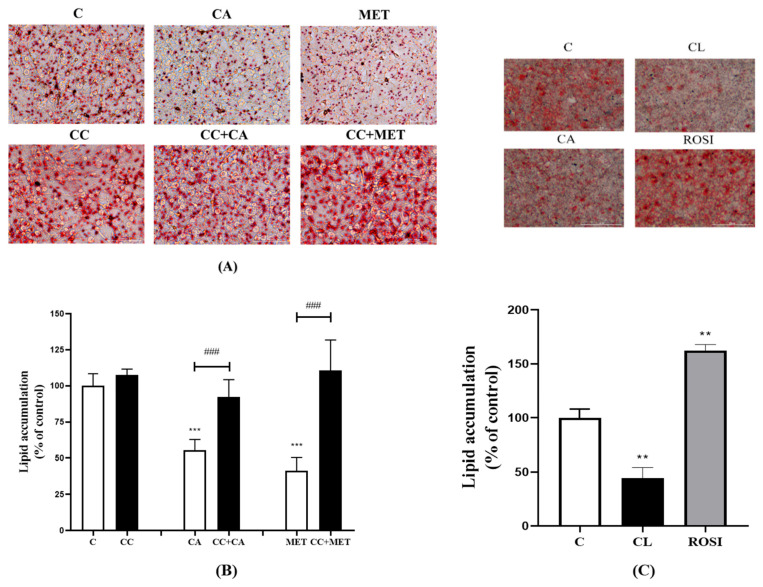
Effects of CA on 3T3-L1 adipocyte lipid content. Fully differentiated 3T3-L1 adipocytes were pretreated for 1 h without (C) or with CC (25 mM), followed by a treatment without (C) or with CA (10 μM) or MET (5 mM) for 24 h in serum-deprived media. After treatment: (**A**) The cells were stained with Oil Red O (ORO) and microscopic images were taken using the color field filter on a Cytation Gen5 multimode imaging microscope (×20). (**B**) Oil Red O was extracted from the cells and the intensity of the supernatant was measured at 490 nm using an ELISA plate reader. (**C**) Fully differentiated 3T3-L1 adipocytes were treated with CA (10 μM), β_3_-adrenergic agonist (CL 316 243) (1 μM), or the PPARγ activator rosiglitazone (ROSI) (10 μM) for 24 h followed by ORO stain and absorbance measurements. The results are the mean ± standard error (SE) of four to six independent experiments, expressed as a percent of the control: ** *p* < 0.01, *** *p* < 0.001 vs. control and ### *p* < 0.001, as indicated.

A reduction in lipid accumulation is correlated with browning and, therefore, serves as a physiological relevant marker of browning. To assess the lipid content of the 3T3-L1 adipocytes, we stained 3T3-L1 adipocytes with ORO, a lipophilic dye that binds lipids and colors them red. The intensity of the red is directly proportionate to the amount of lipid accumulated in the cells. The treatment of 3T3-L1 adipocytes with 10 μM CA significantly reduced the accumulation of lipids (55.56 ± 3.01% of control, *p* < 0.001). This lipid-lowering effect of CA is comparable to that of a treatment with 5 mM MET (41.38 ± 4.09% of control, *p* < 0.001) (Figure 1A,B), a drug derived from the plant French Lilac (Galega Officinalis), used as the first line in a T2DM treatment and known to exhibit lipid-lowering effects in vitro and in vivo [22,23]. Furthermore, the CA- and MET-induced reduction in lipid accumulation was abolished in the presence of the AMPK inhibitor compound C (CC) (92.26 ± 5.38% and 110.6. ± 11.47% of the control, respectively; both *p* < 0.001) (Figure 1A,B). We wished to compare the effects of CA with the effects of another established lipid-lowering agent/chemical and, for this reason, we used the β_3_-adrenergic agonist CL 316 243, known to exhibit potent lipid-lowering effects and act as a browning agent [24]. As expected, the treatment of 3T3-L1 adipocytes with 1 μM CL significantly lowered the lipid content (44.53 ± 9.65% of control, *p* < 0.01). The effect of CA was comparable to the effect achieved with the CL (Figure 1C) and MET treatments (Figure 1A,B). In addition, we used rosiglitazone (ROSI), a thiazolidinedione drug and a PPARγ agonist that was previously shown to increase lipid accumulation in adipocytes [25]. The treatment with ROSI increased the levels of lipids (162 ± 5.65% of control, *p* < 0.01) (Figure 1C). Overall, these data indicate that CA, similar to CL and MET, has lipid-lowering effects in 3T3-L1 adipocytes.

### 3.2. CA Increases Mitochondrial Density

**Figure 2:**
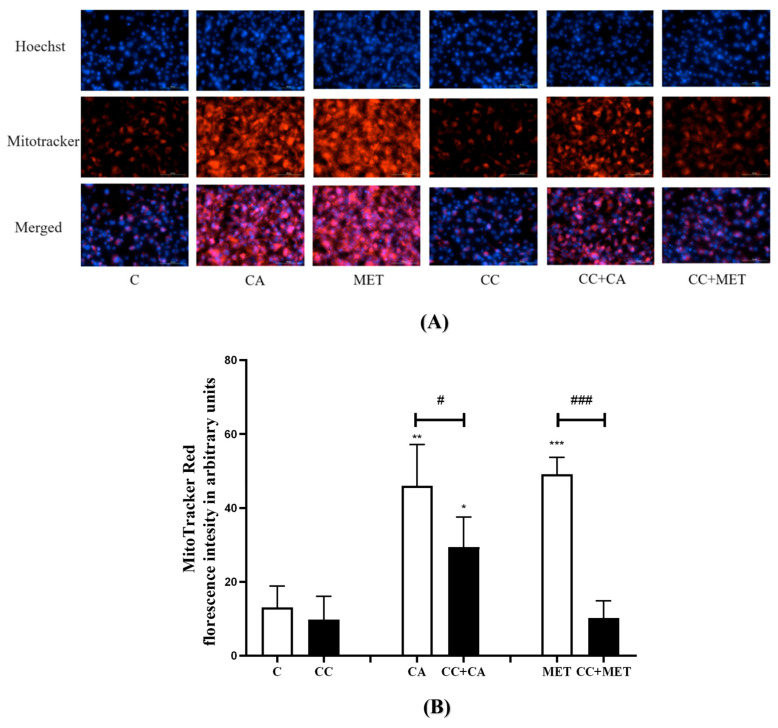
Effects of CA on mitochondrial density: Fully differentiated 3T3-L1 adipocytes were treated without (C) or with CA (10 μM) in the presence or absence of CC for 24 h followed by exposure to 250 nM MitoTracker reagent and 2.5 mg/mL Hoechst blue for 30 min. The cells were then fixed and visualized with Cytation5 using TexasRed (abs/em 644/665 nm) and DAPI/Hoechst filter. Pictures of the plate were taken automatically at the same time using the Cytation5 recommended protocol using the Hoechst/DAPI filter to detect the nuclei (**A**). The intensity of the red florescence was expressed in arbitrary units (**B**). Hoechst blue images were merged with the MitoTracker Red and pictures were created. The data are the mean ± SE of five to six separate experiments. * *p* < 0.05, ** *p* < 0.01, *** *p* < 0.001 vs. control, # *p* < 0.05, and ### *p* < 0.001.

Next, we investigated the effects of CA on mitochondrial density, as the browning of WAT is known to increase mitochondrial biogenesis and activity [26]. To assess the effect of CA on mitochondrial density, we used MitoTracker Deep Red, a dye that accumulates in active mitochondria and fluoresces red upon excitation; thus, we could visualize active mitochondria. Our results show that the treatment with CA significantly increased the mitochondrial density (46.04 ± 4.96, *p* < 0.01) relative to the control (13 ± 2.36) (Figure 2), an effect that was comparable to that of the MET treatment (49.12 ± 2.04, *p* < 0.001).

Furthermore, the exposure of the cells to CC attenuated the CA-induced increase in mitochondrial biogenesis (−37%, *p* < 0.05), indicating that the CA-mediated effects on mitochondrial density are at least in part mediated by AMPK. Similarly, the MET-induced increase in mitochondrial density was significantly diminished by the treatment with CC (−80% ± 2.02, *p* < 0.001). Additionally, we used Hoechst stain to visualize the nuclei of the cells and to assess the overall morphology, double-check cell viability, and account for any differences in cell number. Our results show that there were no significant differences in the number of nuclei across the treatment groups, indicating no cytotoxicity from the treatment and further confirming no effect (reduction) in cell viability (Figure 2). The number of cells were automatically counted by Cytation 5.

### 3.3. CA Activates AMPK in 3T3-L1 Adipocytes

**Figure 3:**
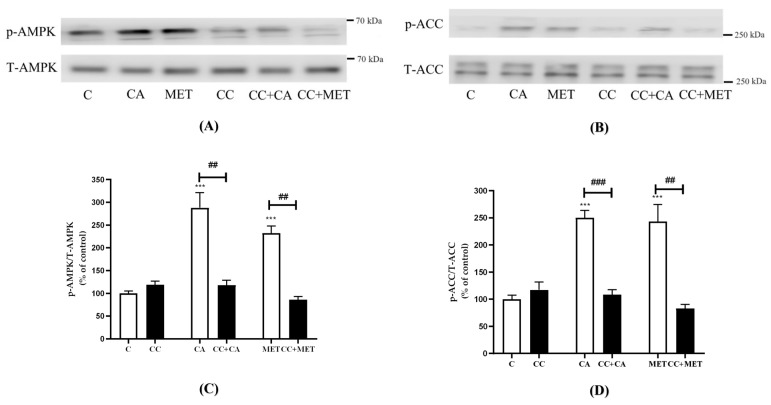
CA and MET increase AMPK and ACC phosphorylation in 3T3-L1 adipocytes. Fully differentiated 3T3-L1 adipocytes were incubated without (C) or with carnosic acid (CA) (10 μM) or metformin (MET) (5 mM) for 24 h in the absence or presence of compound C (CC) (25 μM). After treatment, the cells were lysed and SDS-PAGE was performed, followed by immunoblotting using specific antibodies to recognize the total and phosphorylated (Thr172) levels of AMPK and ACC. Representative blots are shown (**A**,**B**). The densitometry of the bands was measured and expressed in arbitrary units as a percent of the control (**C**,**D**). The data are the mean ± SE of seven to eight separate experiments., *** *p* < 0.001 vs. control, ## *p* < 0.01, ### *p* < 0.001, as indicated.

Figure 3 shows that the treatment of 3T3-L1 adipocytes with CA or metformin (MET; a well-known AMPK activator) significantly increased the phosphorylation of AMPK (CA: 288.0 ± 33.34, MET: 232.3 ± 15.43% of control, *p* < 0.001) and ACC (CA: 250.2 ± 13.43% and MET: 243.4 ± 31.18% of the control, both *p* < 0.001). Importantly, CA- and MET-induced AMPK phosphorylation was completely prevented by compound C (CC) (CC+CA, 106.3 ± 3.16, CC+MET: 72.4 ± 4.7% of the control), which is a well-known AMPK inhibitor (Figure 3). Furthermore, the CA- and MET-induced phosphorylation of ACC, the downstream target of AMPK, was attenuated by the CC treatment (CC+CA: 108.5 ± 9.2% and CC+MET: 83 ± 7.2% of the control) (Figure 3), further indicating the effective blockage of AMPK activation by CC.

### 3.4. CA Increases UCP1 Content in 3T3-L1 White Adipocytes

**Figure 4:**
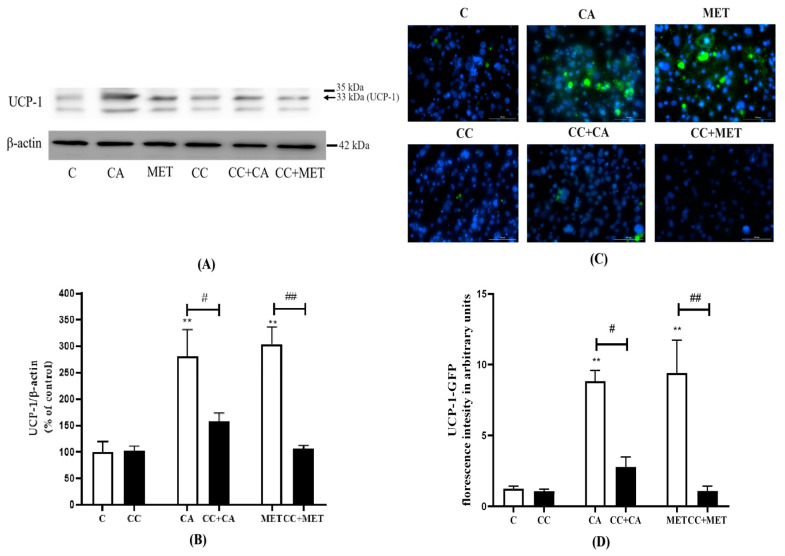
Effects of CA on UCP-1 levels. Fully differentiated 3T3-L1 adipocytes were pretreated for 1 h without (C) or with CC (25 mM), followed by treatment without (C) or with CA (10 μM) or MET (5 mM) for 24 h in serum-deprived media. After treatment, the cells are lysed and SDS-PAGE was performed followed by immunoblotting using specific antibodies to recognize the total levels of UCP-1 or β-actin and immunostaining using an anti-UCP-1 primary antibody and AlexaFluor488 secondary antibody. Hoechst blue stain was used to label the nuclei. Representative blots are shown (**A**). The densitometry of the bands was measured and expressed in arbitrary units as a percent of the control (**B**). Images were taken with Cytation5, a florescence microscope using Green Florescent Protein (GFP) and DAPI/Hoechst filter (**C**). The intensity of the green florescence was measured using ImageJ and is expressed in arbitrary units (**D**). The data are the mean ± SE of six to eleven separate experiments. ** *p* < 0.01 vs. control, # *p* < 0.05, ## *p* < 0.01, as indicated.

White adipocyte browning is correlated with an increase in brown adipocyte marker expression; thus, we next investigated the effects of CA on the established browning marker UCP-1. The treatment with CA markedly increased the expression of UCP-1 (280.7 ± 50.84% of the control, *p* < 0.01) to levels comparable to those of MET (304 ± 32.72% of control, *p* < 0.01) (Figure 4A,B). Exposure to CC significantly attenuated the CA-induced effects on UCP-1 protein levels (−44%, *p* < 0.05). The MET-induced increase in UCP-1 levels was also blocked by CC (−66%, *p* < 0.01) (Figure 4A,B). In addition to Western blotting, we examined UCP-1 expression by immunocytochemistry using UCP-1 primary antibodies that were detected by AlexaFlour488, a green florescence antibody. Our immunocytochemistry data are in agreement with our Western blot data, indicating that both CA (8.84 ± 0.76, *p* < 0.01) and MET (9.40 ± 2.32, *p* < 0.01) increased the expression of UCP-1 relative to the untreated control (1.25 ± 0.17) and that the treatment with CC reduced these responses (CC+CA: −69%, CC+MET: −89%, *p* < 0.05 and *p* < 0.01, respectively) (Figure 2 and Figure 4C).

### 3.5. CA Increases the Expression of Browning and Mitochondrial Biogenesis Markers

**Figure 5:**
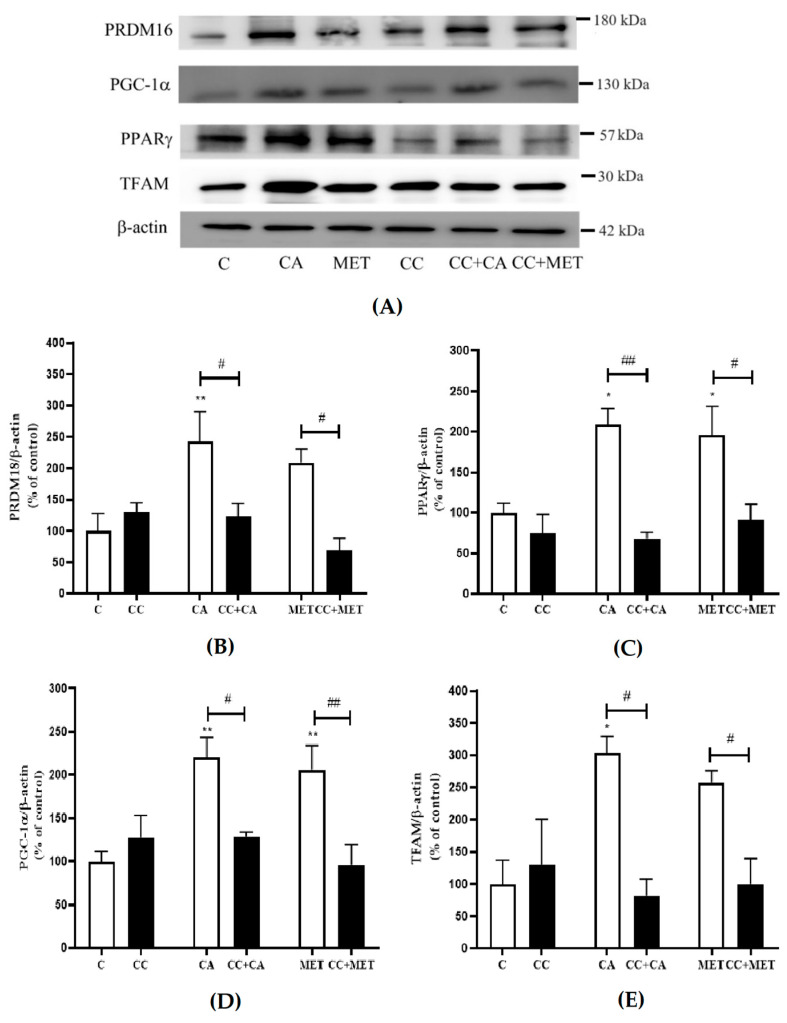
Effects of CA on browning markers: Fully differentiated 3T3-L1 adipocytes were incubated without (C) or with CA (10 μM) or MET (5 mM) for 24 h in the absence and presence of CC (25 μM). After treatment, the cells are lysed and SDS-PAGE was performed, which was followed by immunoblotting using specific antibodies to recognize the total levels of PPARγ, PRDM18, PGC-1α, TFAM, or β-actin. Representative blots are shown (**A**). The densitometry of the bands was measured and is expressed in arbitrary units as a percent of the control (**B**–**E**). The data are the mean ± SE of seven to nine separate experiments. * *p* < 0.05, ** *p* < 0.01 vs. control, # *p* < 0.05, and ## *p* < 0.01, as indicated.

The process of browning involves increases in thermogenic protein expression, such as PRDM16, PPARγ, PGC-1α, and TFAM [27]. To further investigate the effects of CA on adipocyte browning, we examined its effects on these well-established browning and mitochondrial biogenesis markers. The treatment with CA significantly increased the expression of major browning proteins (PRDM16: 242 ± 47.39, PPARγ: 176.7 ± 15.40, PGC-1α: 220 ± 23.04 and TFAM: 303.1 ± 25.79% of the control, *p* < 0.05 and *p* < 0.01) to levels comparable to those of metformin (PRDM: 208.6 ± 21.76, PPARγ: 195.6 ± 35.85, PGC-1α: 206.3 ± 27.87, and TFAM (257.6 ± 18.01% of the control, *p* < 0.05 and *p* < 0.01) (Figure 5A–E). The treatment with CC significantly attenuated the CA-induced effects on browning and mitochondrial biogenesis markers (PRDM16: −50%, PPARγ: −62%, PGC-1α: −22%, and TFAM: −73%, *p* < 0.05 and *p* < 0.01). Similarly to CA, the effects of MET were also reduced by CC (PRDM16: −67%, PPARγ: −54%, PGC-1α: −54%, and TFAM: −62%, *p* < 0.05 and *p* < 0.01) in agreement with other studies [28]. These data indicate that AMPK may be involved in the CA-induced increase in browning and mitochondrial biogenesis markers (Figure 5A–E).

### 3.6. Carnosic Acid (CA) and the AMPK Inhibitor CC Have No Effect on 3T3-L1 Adipocyte Viability

**Figure 6:**
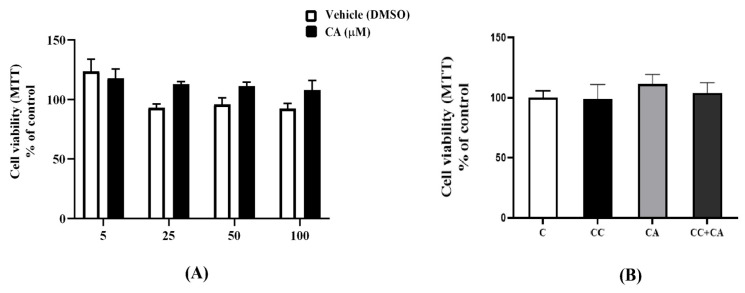
Effects of CA and CC on 3T3-L1 adipocyte viability. (**A**) Fully differentiated adipocytes were treated without (C) or with a range of CA concentrations (5 to 100 μM) or with their corresponding vehicle (DMSO) concentrations for 24 h followed by incubation with MTT. The formazan dye was then solubilized, and absorbance was measured at 570 nm. Cell viability is expressed as a percent of the control (C) untreated cells (**B**) Fully differentiated adipocytes were treated without (C) or with CA in the absence or presence of CC for 24 h followed by MTT assay. The dye was solubilized and read at 570 nm. The values were expressed as a percent of the control and are the mean ± SEM of three independent experiments.

Next, we wished to examine if the observed lipid-lowering effects of CA were due to cell death, and we utilized an MTT viability assay. We exposed cells to various concentrations of CA (5, 25, 50, and 100 μM) and DMSO (CA solvent/vehicle) to match the DMSO levels in the CA-treated cells. The corresponding DMSO concentrations for the CA values of 5, 25, 50, and 100 μM were 0.005, 0.025, 0.05, and 0.1%. The treatment with CA or DMSO did not affect the cell viability (Figure 6A); thus, we are confident that the lipid-lowering effects of CA were not due to cell injury or cell death. The concentration of 0.1% DMSO corresponding to the treatment with 100 μM CA did not have significant effects on adipocyte viability. Additionally, we investigated the effects of CC on cell viability, as reports indicate that CC may stress the cells and cause cell death [29,30]. Our results show that the treatment with CC alone and in combination with CA did not affect 3T3-L1 adipocyte viability in this study (Figure 6B).

### 3.7. CA Inhibits GSK3β

**Figure 7:**
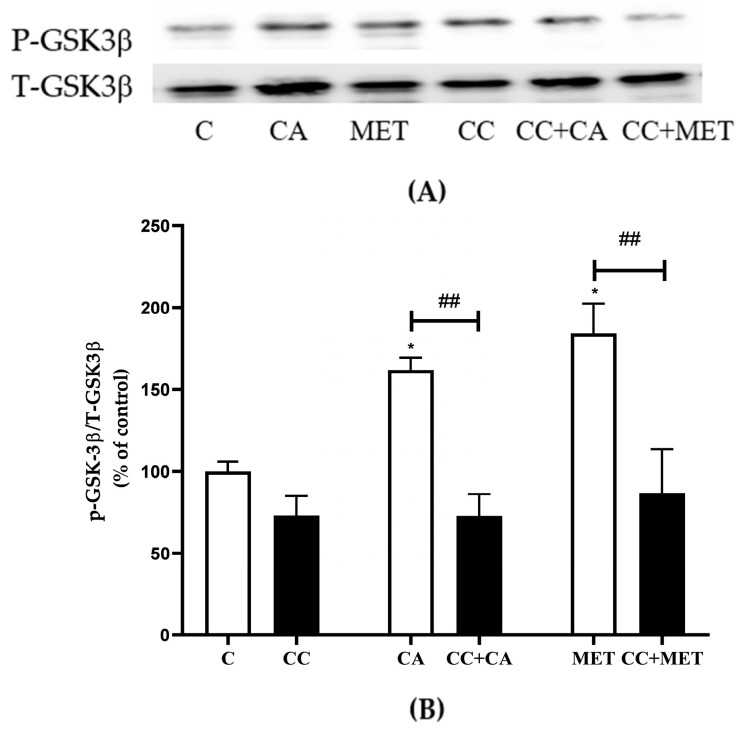
Effects of CA and MET on GSK3β. Fully differentiated 3T3-L1 adipocytes were incubated without (C) or with CA (10 μM) or MET (5 mM) for 24 h in the absence or the presence of CC (25 μM). After the treatment, the cells are lysed and SDS-PAGE was performed, which was followed by immunoblotting using specific antibodies to recognize the total and phosphorylated (Ser9) levels of GSK3β. Representative blots are shown (**A**). The densitometry of the bands was measured and expressed as a percent of control (**B**). The data are the mean ± SE of two separate experiments. * *p* < 0.05, and ## *p* < 0.01, as indicated.

In addition to AMPK, studies have shown that the inhibition of the constitutively active GSK3β increases thermogenic/browning markers [10]. Based on this evidence, we examined the effects of CA on total and phosphorylated Ser9 GSK3β levels. The phosphorylation of GSK3β on Ser9 is associated with the inhibition of GSK3 activity [31]. Our data show that both CA and MET increased the phosphorylation of Ser9 on GSK3β CA: 162 ± 7.47, MET: 184.0 ± 7.47% of the control). Exposure to CC significantly attenuated the CA- and MET-induced effects on GSK3 phosphorylation levels (CA: 73 ± 13.27% and MET: 183 ± 18.19% of the control, *p* < 0.05, respectively) (Figure 7A,B).

## 4. Discussion

Strategies that induce adipose tissue browning are promising approaches in the prevention and management of obesity and metabolic diseases [32,33]. Polyphenols have been shown to promote browning and, therefore, offer new intervention strategies in the prevention and management of obesity and its related diseases [34,35]. In the present study, using 3T3-L1 adipocytes, we found that CA significantly increased the expression of markers of browning (UCP-1, PRDM16, and PPARγ) and mitochondrial biogenesis (PGC-1α, TFAM, and mitochondrial density). These effects were mediated in part by an activation of AMPK as well as the inhibition of GSK3. Adding physiological relevance to our work, the increased presence of mitochondrial browning markers and biogenesis was met with a significant lipid-lowering effect—which is ultimately consistent with the energy-dissipating function of UCP-1. Moreover, many of these effects of CA were similar to those of metformin (MET), which is well known to induce browning and mitochondrial biogenesis in and have a lipid-lowering effect on fat cells via AMPK activation [23,36,37,38].

The treatment of 3T3-L1 adipocytes with CA showed a significant increase in the UCP-1 protein levels, an established marker of browning, which was also accompanied by elevated levels of PRDM16 and PPARγ. These effects of CA were similar to the effects of MET. Previous studies have shown that the exposure of 3T3L1 adipocytes to 5 mM MET increased UCP-1, PRDM16, and PPARγ [39]. PRDM16 stimulates brown adipogenesis by binding to PPARγ and increasing its transcriptional activity, thus facilitating browning [40]. PPARγ is highly expressed in both brown and white adipocytes and plays a pivotal role in browning as it is involved in causing a significant increase in the expression of brown fat-specific genes, such as UCP1, PGC-1a, TFAM, and PRDM16 [41]. A limitation of this study includes the lack of functional data to fully confirm the presence of beige adipocytes, as respiration and oxygen consumption rates were not measured.

Increased mitochondrial density is a typical feature of the browning of fat tissue [42]. Our data demonstrate that the treatment of 3T3-L1 adipocytes with CA increased the PGC-1α protein levels, a master regulator of mitochondrial biogenesis, and TFAM, a mitochondrial DNA transcription factor, and these effects were comparable to the treatment with MET. Other studies have shown that MET increases PGC-1 and TFAM expression in 3T3-L1 and the brown adipocytes of mice [25,39]. Furthermore, we used MitoTracker and found that CA increased mitochondrial biogenesis in the cells. A caveat for using immunofluorescence labeling, such as GFP-labeled antibodies and MitoTracker, is the potential interference of the lipid droplets with the light emission [43]. This may lead to light infractions and potentially misleading results. However, in our study, we utilized different approaches, and our MitoTracker data are in agreement with our Western blotting data, showing increased UCP-1 protein levels and collectively indicating increased mitochondrial biogenesis.

Our data show that CA phosphorylated AMPK and its downstream target ACC, which is an established readout of AMPK activity, to levels comparable to those of MET. We found that the treatment with CC, an AMPK inhibitor, abolished the CA- and MET-induced phosphorylation of AMPK and ACC, indicating that, in our experiments, CC was successful in blocking AMPK activation. The lipid-lowering effects of CA were significantly attenuated in the presence of CC, indicating that AMPK is involved in the CA-mediated effects. The lipid-lowering effects of CA were comparable to those of MET. MET, at concentrations similar to the ones used in the present study (5 mM), was previously reported by others [23,36] to exhibit strong lipid-lowering effects in 3T3-L1 and primary rat adipocytes in vitro. The similar lipid-lowering effects of MET were shown in vivo. C57BL/6J mice treated with MET (50 mg/bw/day) for 14 weeks had reduced adipocyte size, visceral and inguinal WAT mass, and overall bodyweight by approximately 15.21% [39]. Overall, our findings indicate potent lipid-lowering effects of CA that are similar to those of MET; however, future studies are needed to determine whether these effects can be maintained in vivo.

**Figure 8:**
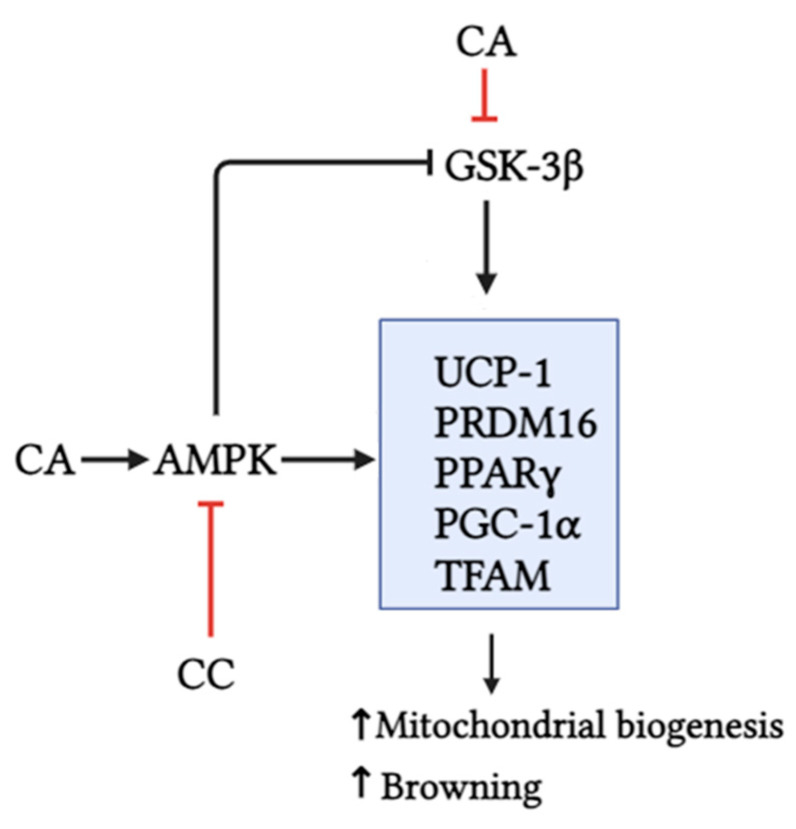
CA activated AMPK, inhibited GSK3β, and increased the expression of browning (UCP-1, PRDM16, and PPARγ) and mitochondrial biogenesis protein markers (PGC-1α and TFAM). Use of compound C, an AMPK inhibitor, significantly attenuated the effects of CA. Created with BioRender.com.

We also found the inhibition of GSK3 with the CA treatment. This is important as GSK3 has been identified as a negative regulator of brown adipocyte function and WAT browning [10]. This inhibition of GSK3 with CA is consistent with previously published results in the cardiac muscle [44] and with unpublished data from our own lab in L6 muscle cells, whereby the CA treatment was found to increase the Ser9 phosphorylation of GSK3. Phosphorylation at this site serves to inactivate GSK3 by preventing substrate recognition. While previous studies have shown that AMPK can inhibit GSK3, whether this inhibition of GSK3 is dependent or independent of the activation of AMPK observed with CA treatment remains unknown. Regardless, we propose that the inhibition of GSK3 and the activation of AMPK can work together to induce a browning phenotype in 3T3L1 cells, ultimately causing the lipid-lowering effect (Figure 8).

## 5. Conclusions

This study demonstrates that the treatment of 3T3-L1 adipocytes with carnosic acid (CA) activated AMPK, inhibited GSK3β, reduced lipid accumulation, and increased browning (UCP-1, PRDM16, and PPARγ) and mitochondrial biogenesis (PGC1α and TFAM) markers (Figure 8). These effects were comparable to those of MET, the first line of T2DM treatment. The use of CC, an AMPK inhibitor, shown in our studies to inhibit AMPK phosphorylation/activation, significantly attenuated the effects of CA (Figure 8). Although these data suggest an important role of AMPK in the CA-mediated effects, future in vitro experiments utilizing AMPK downregulation/knockout approaches, such as small interference RNA, are required to strengthen this evidence. In addition, animal studies and human clinical trials are required to fully examine the in vivo effects of CA in adipocyte browning.

## Data Availability

The original contributions presented in the study are included in the article, further inquiries can be directed to the corresponding author. The raw data supporting the conclusions of this article will be made available by the corresponding author on request.
